# A post-implementation evaluation of BioFire FilmArray Meningitis/Encephalitis panel for pathogen detection in cerebrospinal fluid with a special focus on clinical significance of HHV-6

**DOI:** 10.1128/spectrum.00620-25

**Published:** 2025-11-28

**Authors:** Manoshi Perera, Hemalatha Varadhan, Aileen Oon

**Affiliations:** 1 Microbiology, NSW Health Pathology, John Hunter Hospital 441551 , Newcastle, NSW, Australia; 2 University of Newcastle 5982, Newcastle, NSW, Australia; The George Washington University School of Medicine and Health Sciences, Washington, DC, USA

**Keywords:** FilmArray ME panel, CNS infection, herpesvirus, diagnostic NAAT, viral meningitis

## Abstract

Syndromic molecular testing for the diagnosis of infections has been advantageous given high sensitivity and rapid diagnostics; however, challenges remain with appropriate interpretation. A post-implementation evaluation of the cerebrospinal fluid (CSF) BioMerieux BioFire FilmArray Meningitis/Encephalitis panel in an adult and pediatric population was performed. The aims were to assess the prevalence and epidemiology of all targets and to correlate laboratory parameters with results from human herpesvirus 6 (HHV-6) detection in CSF to identify opportunities to apply diagnostic stewardship. A retrospective observational study was conducted on CSF Nucleic Acid Amplification Test (NAAT) results performed at a referral laboratory in Australia between 19 March 2022 and 31 July 2023. Laboratory data were extracted, with additional clinical data obtained for the HHV-6 positive subcohort. Of 1,490 CSF specimens identified, 287 (19.3%) had a positive NAAT. Non-neonate CSF specimens with white cell count (WCC) above the normal range had a higher proportion of NAAT-positive rates (35.3%) compared to those with WCC < 5 × 10^6^ cells/L (6.5%) (*P* < 0.001). A positive NAAT result was detected in 90 (39.1%) specimens in neonates, with enterovirus and parechovirus detected most frequently. Concordance of bacterial and fungal targets and culture results was seen in 16 (33.3%) specimens. Forty-eight CSF specimens had HHV-6 detected, with a median age of 24.3 years (interquartile range: 0.5–58.7) at collection and 10 specimens with a documented immunocompromised status. This study highlights the caution required when utilizing syndromic diagnostic assays for the detection of CSF pathogens without associated diagnostic stewardship strategies and the potential utility of CSF WCC as a criterion for further review.

**IMPORTANCE:**

Utilization of molecular testing methods enables rapid and high-sensitivity diagnostics, which can have significant impacts upon clinical outcomes in meningitis and encephalitis. However, challenges remain with the interpretation of results when syndromic panels are used, such as with the detection of human herpesvirus 6 (HHV-6) in cerebrospinal fluid (CSF). This study aimed to evaluate the BioMerieux BioFire FilmArray Meningitis/Encephalitis panel for the detection of pathogens in CSF in an adult and pediatric population. Data demonstrated that performing CSF nucleic acid amplification testing without diagnostic stewardship strategies in place can be associated with increased detection of potential bystander pathogens, including HHV-6.

## INTRODUCTION

The introduction of syndromic molecular testing methods for the diagnosis of central nervous system (CNS) infections has been advantageous, given high sensitivity and subsequent implications of rapid diagnostics leading to prompt antimicrobial rationalization, optimal clinical outcomes, and an impact upon financial burden (1).

Global epidemiology studies have identified a comparatively low rate of CNS infection in Australia, with an incidence of encephalitis of 2.08 per 100,000 person-years in 2019 (2), and an incidence of meningitis in 2016 of 0.5 per 100,000 persons (3). A large prospective cohort study identified enteroviruses, human parechovirus, herpes simplex virus (HSV), and *Streptococcus* species as the highest causes of meningoencephalitis among Australian children (4). Similarly, in a study over an 18-year period, HSV and varicella zoster virus (VZV) were identified as the most common causes of hospitalization due to encephalitis among adults. The etiology of CNS infections has likely also been impacted by the implementation of nationwide targeted immunization schedules for *Streptococcus pneumoniae*, *Haemophilus influenzae*, and *Neisseria meningitidis* (5).

Human herpesvirus 6 (HHV-6) is a ubiquitous herpesvirus with a wide spectrum of disease from benign, self-limiting illness in children to encephalitis within an immunocompromised host (6). The seroprevalence of HHV-6 in the adult population globally is estimated to be >90%, with the majority of primary infections occurring in childhood (7). HHV-6 has a unique ability among other members of the family Herpesviridae to integrate into the subtelomeric regions of human chromosomes in every host cell (6). Described as chromosomally integrated HHV-6 (ciHHV-6), it has an estimated prevalence of 1% in humans (8). Hence, detection of HHV-6 nucleic acid in cerebrospinal fluid (CSF) may represent primary infection, latency, secondary reactivation, or ciHHV-6 present with host cells within CSF (1). Subsequently, challenges arise in the interpretation of results from a highly sensitive assay like the BioMerieux BioFire FilmArray Meningitis/Encephalitis (FAME) assay and necessitate appropriate correlation with clinical findings (9).

No standardized treatment guidelines are available for the management of HHV-6; however, literature has reported positive outcomes with the use of ganciclovir, foscarnet, and less frequently, cidofovir (10, 11). These antiviral therapies are associated with significant adverse effects, including anemia, electrolyte disturbance, and nephrotoxicity, and hence require close monitoring during use (10). Subsequently, the implications of reporting and interpretation of results need to be carefully considered by laboratories that perform diagnostic testing.

In March 2022, our laboratory implemented the BioMerieux BioFire FAME assay as part of CSF multiplex Nucleic Acid Amplification Test (NAAT) testing in order to facilitate more rapid diagnostics for a large, heterogeneous adult and pediatric patient population. Prior to the implementation of the assay in March 2022, CSF specimens were referred for testing of targeted pathogens as specifically requested by the clinician.

### Aim

Evaluation of the diagnostic performance of the BioMerieux BioFire FAME panel (from this point forward referred to as CSF NAAT), on CSF in a population of adult and pediatric patients was undertaken with two main aims. The first is to assess the prevalence and epidemiology of all NAAT targets, and the second is to correlate laboratory parameters with results from HHV-6 detection on CSF to identify opportunities for the laboratory to apply diagnostic stewardship.

## MATERIALS AND METHODS

A retrospective observational study was conducted of CSF NAAT results, performed at a single referral laboratory in Newcastle (New South Wales), Australia between 19 March 2022 and 31 July 2023. The laboratory service performs testing for regional and rural health facilities in the region and is co-located with an adult and a specialist children’s hospital, which includes transplant, hematology, and oncology clinical services. Inclusion criteria included pediatric and adult CSF specimens obtained from lumbar puncture, at least 200 µL volume collected, and referred specimens from Hunter New England and Mid-North Coast Local Health Districts. Exclusion criteria included forensic specimens, repeat specimens collected from the same patient during the same admission period, and specimens collected external to the pre-defined local health districts.

### Laboratory procedures

CSF multiplex NAAT testing is ordered at clinician discretion, and the BioMerieux BioFire FAME assay is performed as per the manufacturer’s instructions for use. The CSF NAAT is an automated, real-time assay from BioMerieux BioFire Diagnostics which enables the isolation, amplification, and detection of nucleic acid from seven viral targets—HSV 1 and 2, VZV, HHV-6, human parechovirus, enterovirus, and cytomegalovirus (CMV)—as well as *Cryptococcus neoformans/gattii* and six bacterial targets—*Listeria monocytogenes*, *Neisseria meningitidis*, *Streptococcus pneumoniae*, *Streptococcus agalactiae*, *Escherichia coli* K1, and *Haemophilus influenzae*. Analysis occurs simultaneously from a single 200 µL CSF specimen, producing a qualitative result within approximately 1 h. Detection of a pathogen is considered a critical result and is verbally notified to the treating clinician.

CSF microscopy and bacterial culture are performed onsite as per local laboratory standard procedures. Microscopy, including cell count and Gram stain, is performed manually, and culture plates are incubated for a minimum of 5 days. Organism identification is performed using MALDI-ToF (Bruker Biotyper).

HHV-6 serum quantitative NAAT and serology were referred to an external reference laboratory. No other targeted NAAT testing was performed on CSF specimens.

### Data collection

Laboratory data were extracted from the laboratory information system (AUSLAB). This included results of CSF NAAT, CSF culture, and CSF microscopy (white cell count, WCC), in addition to patient demographics of age and gender. Additional laboratory data were obtained for the CSF HHV-6 positive subcohort, including CSF microscopy (red blood cell, RBC; WCC differential), CSF biochemistry (glucose, lactate, and protein), peripheral WCC and differential, serum HHV-6 viral load, and HHV-6 serology. Additional clinical details, including immunocompromised status, documented clinical diagnosis, and antiviral therapy received, were obtained for the CSF HHV-6 positive subcohort.

Normal CSF WCC in adults and children (aged between 1 month and 18 years) was defined as <5 × 10^6^ cells/L and RBC as <5 × 10^6^ cells/L (12, 13). Normal CSF WCC in neonates (≤1 month) was defined as <20 × 10^6^ cells/L (14, 15). Normal ranges for CSF biochemistry were defined as follows: glucose 2.8–4.5 mmol/L with lower limit of normal (LLN) < 2.8 mmol/L, protein 0.2–0.7g/L with upper limit of normal (ULN) > 0.7 g/L, and lactate < 2.8 mmol/L with ULN ≥ 2.8 mmol/L (13). Normal CSF biochemistry in neonates included glucose ≥ 2.0 mmol/L and protein < 1.0 g/L (12).

### Statistical analysis

Standard descriptive statistics were used to analyze participant demographics, microbiological data, and clinical data. Qualitative/categorical variables were described as counts and proportions and compared with the chi-square test. Statistical significance was accepted as *P* < 0.05. Microsoft Excel version 16.0 and IBM SPSS version 29.0 were used for statistical analysis.

## RESULTS

### Total cohort

#### Overview

During the study period, 1,490 CSF specimens were identified from 1,457 different patients. Thirty-three patients had more than one CSF NAAT performed in the study period during different admission episodes, with the median number of days between collection being 25 (interquartile range [IQR]: 17.0–65.0). There was a male preponderance of 52% with the median age at CSF collection of 34.9 years (range 0.3–61.7 years). The cohort comprised 958 (64.3%) specimens from adults, 302 (20.3%) from children, and 230 (15.4%) from neonates.

#### CSF NAAT

**Fig 1:**
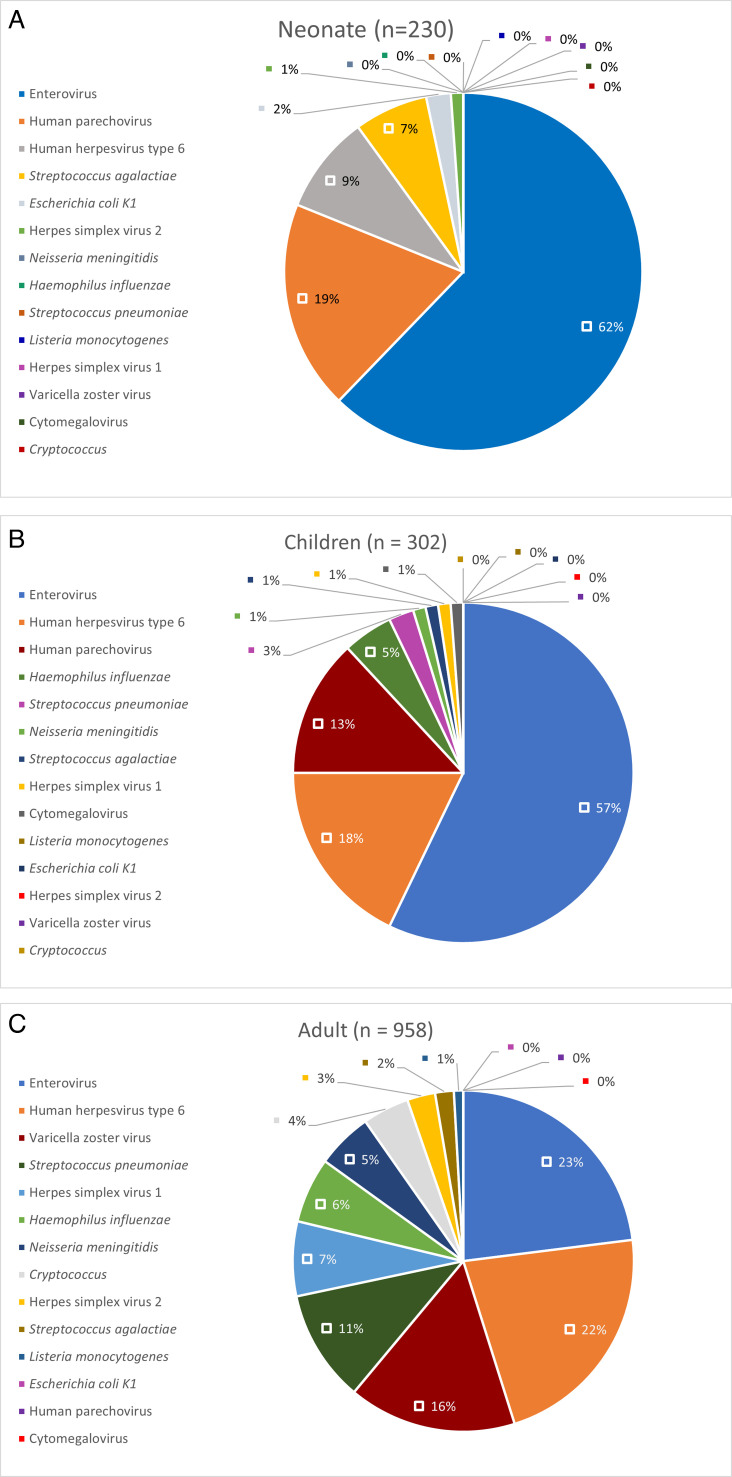
(**A**) Proportion of positive CSF NAAT panel targets for the subcohort of neonates (≤1 month). (**B**) Proportion of positive CSF NAAT panel targets for the subcohort of children (between 1 month and 18 years). (**C**) Proportion of positive CSF NAAT panel targets for the subcohort of adults (≥18 years).

A positive CSF NAAT was identified in 287 specimens (19.3%), with five specimens (0.3%) having more than one organism detected. This included one specimen with VZV and *Streptococcus pneumoniae* detected, two specimens with HHV-6 and enterovirus, one specimen with HHV-6 and *Streptococcus agalactiae*, and one specimen with HHV-6 and VZV (Table 1). Enterovirus was detected in 130 specimens (8.7%), and HHV-6 was detected in 48 specimens (3.2%). There was a variable distribution of pathogens detected between the neonate, children, and adult subcohorts (Fig. 1).

**TABLE 1:** Patient characteristics and CSF NAAT results^*a*^

Characteristic	*n* = 1,490 (%)
Male	775 (52)
Female	715 (48)
Age at CSF collection, years (median, IQR)	34.9 (0.3–61.7)
Neonate	230 (15.4)
Children	302 (20.3)
Adult	958 (64.3)
Positive CSF NAAT	287 (19.3)
>1 detected	5 (0.3)
Enterovirus RNA detected	130 (8.7)
Human herpesvirus type 6 DNA detected	48 (3.2)
Human parechovirus RNA detected	28 (1.9)
Varicella zoster virus DNA detected	18 (1.2)
*Streptococcus pneumoniae* DNA detected	14 (0.9)
*Haemophilus influenzae* DNA detected	11 (0.7)
Herpes simplex virus type 1 DNA detected	9 (0.6)
*Streptococcus agalactiae* DNA detected	9 (0.6)
*Neisseria meningitidis* DNA detected	7 (0.5)
*Cryptococcus* DNA detected	5 (0.3)
Herpes simplex virus type 2 DNA detected	4 (0.3)
*Escherichia coli* K1 DNA detected	2 (0.1)
Cytomegalovirus DNA detected	1 (0.1)
*Listeria monocytogenes* DNA detected	1 (0.1)

^*a*^ CSF, cerebrospinal fluid; DNA, deoxyribonucleic acid; IQR, interquartile range; RNA, ribonucleic acid.

#### CSF culture

There was no growth from culture in 1,421 (95.4%) specimens, and culture was not performed for 15 (1.0%) specimens. Of samples which isolated an organism from culture, 38 (70.4%) were not target pathogens within the CSF NAAT panel—these included coagulase-negative staphylococci (20, 37%), *Candida dubliniensis* (1, 1.9%), *Staphylococcus aureus* (1, 1.9%), other Gram-positive organisms (13, 24.1%), and other Gram-negative organisms (3, 5.6%). Concordance of bacterial and fungal NAAT targets and culture results was seen in 16 (33.3%) specimens, with concordance seen in all *Cryptococcus* cases (Table 2). Four CSF specimens with a virus detected on NAAT isolated bacteria on CSF culture, including coagulase-negative staphylococci and other Gram-positive organisms.

**TABLE 2:** Concordance of CSF NAAT and culture results^*a*^^,^^*b*^

Pathogen	Positive CSF culture	Positive NAAT	% (positive CSF culture/ positive NAAT)
*Cryptococcus* spp.			
*Cryptococcus neoformans*	3	3	100
*Cryptococcus gattii*	2	2	100
*Haemophilus influenzae*	4	11	36.4
*Streptococcus pneumoniae*	5	14	35.7
*Streptococcus agalactiae*	2	9	22.2
*Escherichia coli*	0	2	0
*Neisseria meningitidis*	0	7	0

^*a*^ CSF, cerebrospinal fluid; NAAT, Nucleic Acid Amplification Test.

^*b*^ Exclusion of *L. monocytogenes* CSF specimen where culture was not performed.

#### CSF microscopy

**Fig 2:**
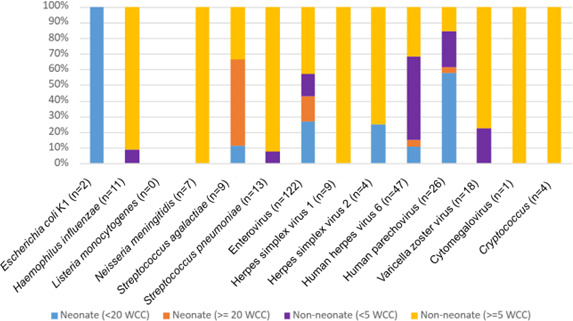
Comparison of positive NAAT targets in patients with elevated CSF WCC (non-neonates ≥ 5 × 10^6^ cells/L, neonates ≥ 20 × 10^6^ cells/L) to patients with a WCC within normal range (non-neonates < 5 × 10^6^ cells/L, neonates < 20 × 10^6^ cells/L). WCC was not recorded for 13 specimens.

The median WCC on CSF microscopy was 3 × 10^6^ cells/L (IQR: 1–16.8), with 61 specimens without documented WCC. A WCC within normal range (<5 × 10^6^ cells/L) was identified in 844 (844/1,260, 67.0%) specimens among non-neonates and 165 (165/230, 71.7%) specimens with normal WCC (<20 × 10^6^ cells/L) in the neonate subcohort. Among all age groups, there were 24 (2.4%) positive CSF cultures with WCC within normal range. The organisms isolated included coagulase-negative staphylococci and other Gram-positive and Gram-negative organisms. A positive CSF NAAT result was obtained in 7.5% of specimens in this cohort with WCC within normal range, with enterovirus most frequently detected (Fig. 2). In contrast, there were 347 CSF specimens from non-neonates with elevated WCC (≥5 × 10^6^ cells/L) and negative CSF culture, of which 115 (33.1%) had a positive NAAT. Overall, non-neonate CSF specimens with a WCC above normal range had a higher proportion of NAAT-positive rates (35.3%) compared to those with a WCC < 5 × 10^6^ cells/L (6.5%) (*P* < 0.001).

### Age-based subcohorts

There were 958 (64.3%) adult CSF specimens and 532 obtained from pediatrics patients, comprising 230 (15.4%) neonates and 302 (20.3%) children, respectively. Of the latter cohort, 16 had growth on respective CSF cultures, including one specimen with *Haemophilus influenzae*, three specimens with *Streptococcus*, one specimen with *Candida*, and the others either coagulase-negative staphylococci or other Gram-positive organisms (Table 3). A positive NAAT result was detected in 90 (39.1%) specimens in neonates and 84 (27.8%) specimens in children, with proportionally viral targets more frequently detected including enterovirus and parechovirus. While enterovirus (23%) and HHV-6 (22%) were the most commonly detected targets among NAAT-positive specimens in adults, there were also more frequent bacterial/fungal detections compared to the pediatric cohort. This was supported by 38 positive CSF cultures, including five *Cryptococcus* isolates, five *Streptococcus pneumoniae*, and three *Haemophilus influenzae*.

**TABLE 3:** CSF NAAT and culture positivity in specimens from neonates and children subcohorts^*a*^

Characteristic	Neonates, *n* = 230 (%)	Children, *n* = 302 (%)
Growth on CSF culture^*b*^	7 (3.0)	9 (3.0)
Positive NAAT	90 (39.1)	84 (27.8)
Enterovirus	56 (24.3)	48 (15.9)
Parechovirus	17 (7.4)	11 (3.6)
HHV-6	8 (3.4)	15 (5.0)
*Streptococcus agalactiae*	6 (2.6)	1 (0.3)
*Haemophilus influenzae*	0 (0)	4 (1.3)
*Listeria monocytogenes*	0 (0)	0 (0)
*Escherichia coli* K1	2 (0.9)	0 (0)
*Streptococcus pneumoniae*	0 (0)	2 (0.7)
*Neisseria meningitidis*	0 (0)	1 (0.3)
CMV	0 (0)	1 (0.3)
HSV 1	0 (0)	1 (0.3)
HSV 2	1 (0.4)	0 (0)
VZV	0 (0)	0 (0)
*Cryptococcus*	0 (0)	0 (0)

^*a*^ CMV, cytomegalovirus; CSF, cerebrospinal fluid; HHV-6, human herpesvirus 6; HSV, herpes simplex virus; NAAT, Nucleic Acid Amplification Test; VZV, varicella zoster virus.

^*b*^ Other—*Bacillus* spp., *Brevibacterium* spp., *Cutibacterium* spp., and *Paenibacillus* spp.

### HHV-6 positive subcohort

Forty-eight (48/1,490) CSF specimens had HHV-6 detected via NAAT, with a male preponderance (58.3%) and a median age of 24.3 years (IQR: 0.5–58.7) at CSF collection. An elevated CSF RBC above the normal range was noted in 41.7% of specimens (Table 4). An immunocompromised status was documented with 10 CSF specimens, of which two underwent HHV-6 quantitative NAAT and serology.

**TABLE 4:** Microbiological and patient characteristics of CSF specimens with HHV-6 detected^*a*^

Characteristic	*n* = 48 (%)
Male	28 (58.3)
Age at CSF collection, years (median, IQR)	24.3 (0.5–58.7)
CSF WCC, ×10^6^ cells/L (median, IQR)^*b*^	2 (0.5–11.5)
CSF WCC differential (median, IQR)	
PMN %	3.5 (0–23.75)
Mononuclear %	82 (36.3–99.5)
Not performed	30 (62.5)
CSF RBC ≥ 5, ×10^6^ cells/L^*b*^	20 (41.7)
Neonate	5 (25.0)
Children	3 (15.0)
Adult	12 (60.0)
CSF biochemistry	
Glucose (<LLN)	7 (14.6)
Protein (>ULN)^*b*^	12 (25.0)
Lactate (>ULN)	2 (4.2)
Not recorded	16 (33.3)
Immunocompetent; peripheral WCC, ×10^9^ cells/L (median, IQR)	9.6 (7.4–13.6)
Lymphocyte %	27 (16–48)
Neutrophil %	62 (34–74)
Immunocompromised; peripheral WCC, ×10^9^ cells/L (median, IQR)	7.6 (4.4–9.7)
Lymphocyte %	12 (9–15)
Neutrophil %	75 (71–88)
Not recorded	6 (12.5)
Serum HHV-6 quantitative NAAT	6 (12.5)
Copies/mL (median, IQR)	2,809 (1,924.3–3,915)
HHV-6 serology	6 (12.5)
Recorded clinical diagnosis	
HHV-6 meningoencephalitis or myelitis	11 (22.9)
Non-meningoencephalitis	30 (62.5)
Not recorded	7 (14.6)
Directed anti-viral therapy	7 (14.6)
Not recorded	7 (14.6)
Immunocompromised status	10 (20.8)
Malignancy	5 (50)
Autoimmune disease on immunosuppressants	4 (40)
Transplant recipient	1 (10)
Not recorded	4 (8.3)

^*a*^ CSF, cerebrospinal fluid; HHV-6, humans herpesvirus 6; IQR, interquartile range; LLN, lower limit of normal range; NAAT, Nucleic Acid Amplification Test; PMN, polymorphonuclear cells; RBC, red blood cell; ULN, upper limit of normal range; WCC, white cell count.

^*b*^ Data for 1 patient not recorded.

## DISCUSSION

This study outlines the epidemiology of CSF NAAT positivity in a heterogeneous adult and pediatric population. Furthermore, it highlights parameters for the application of diagnostic stewardship, as exemplified by the frequency of detection of HHV-6.

The advantages of utilizing a CSF multiplex NAAT are often recognized in cases of aseptic meningitis—CSF specimens with elevated WCC and negative culture (16). Our study had positive identification in 33.1% of these specimens via NAAT, including bacterial pathogens. Enterovirus was the most commonly detected pathogen on NAAT in our study, and this was similarly reported in a recent study as the most common cause of aseptic meningitis in a cohort of pediatric and adult patients (16). The overall concordance between culture and bacterial/fungal targets was lower in our study (33.3%) than previously described by Myint et al., though comparable *H. influenzae* and *N. meningitidis* targets (17). Interestingly, our study had 100% concordance between CSF NAAT and culture results for *Cryptococcus gattii* and *Cryptococcus neoformans*. This contrasts with previous studies which have highlighted a high false-negative rate for the CSF NAAT, particularly among patients on anti-fungal therapy and/or who have a low disease burden (18). Other instances of false negatives have been described in association with HSV 1/2 (19). Hence, recommendations made by our laboratory to repeat CSF collection if clinical suspicion for HSV infection remains. Instances of NAAT bacteria detection with sterile CSF culture were noted; however, an attributable cause was not directly assessed—a limitation of our study. Previously ascribed causes include antimicrobial treatment prior to specimen collection in hospital or community, fastidiousness of organism, and organism concentration at assay limit of detection (16, 20). The majority (80.7%) of CSF specimens in our study had a negative NAAT panel, which is similar to rates reported by Precit et al., who had a comparable patient cohort and size (20). This raises concerns about overutilization of the assay (20), emphasizing the need to rationalize testing algorithms and employ diagnostic stewardship practices (21). A lenient testing criterion can be detrimental in placing an added burden on clinicians with regard to the interpretation of results. Restriction criteria previously explored include CSF WCC within the normal range; however, studies have described that the absence of leukocytosis is not always reliable (10, 22). Important exceptions to consider include specimens from neonates and detection of parechovirus, which is commonly associated with the absence of reactive pleocytosis (15, 23). This was similarly seen in our study with detection of bacterial pathogens, including *S. agalactiae* and *E. coli* K1 target via NAAT in neonates with CSF WCC within normal range. Within our cohort, there was a statistically significant difference in NAAT positivity between non-neonate specimens with normal and elevated CSF WCC. There was only 6.5% NAAT positivity among non-neonates with CSF WCC within normal range, with a minority of targets requiring directed therapy and positive cultures with growth of what were deemed likely contaminants. Most pathogens detected in this subcohort included viruses associated with reactivation but not clinical CNS disease, such as HHV-6 and VZV (1). Of the two positive NAAT bacterial detection, *S. pneumoniae* was deemed clinically unlikely and a possible false positive due to contamination, which has previously been reported by Leber et al. (16). While, detection of *H. influenzae* on NAAT with a sterile culture, may be attributable to antimicrobial treatment prior to specimen collection (17). Thereby supporting the use of this WCC criterion among non-neonate specimens as a prompt for clinician discussion prior to proceeding with CSF NAAT.

Another consideration with the use of multiplex NAAT testing methods is the identification of targets which may not correlate with clinical pathogenicity. This is particularly important with viruses given the possibility of subclinical reactivation, latency, and genomic integration (1). The incidence of reported HHV-6 associated meningoencephalitis or transverse myelitis in the HHV-6 positive cohort (22.9%) was similar to that reported by Pandey et al. (24). However, compared to Pandey et al., which was only of pediatric patients, our study cohort had a median age of 24.3 years, with only 35.7% of CSF specimens from patients aged <18 years (24). This is an important consideration, given HHV-6 meningoencephalitis among immunocompetent adults is reportedly rare, and our study cohort included a majority (79.2%) without an immunocompromised status documented (25). Furthermore, the predominant peripheral neutrophilia among the immunocompetent patients may suggest a non-viral etiology. In contrast, HHV-6 associated with severe clinical outcomes has been described among immunocompromised individuals, with manifestations of encephalitis, hemophagocytic lymphohistiocytosis, long-term neurological sequelae, and mortality (26). Although HHV-6 reactivation can occur in up to two-thirds of hematopoietic stem cell transplant recipients, the prevalence of encephalitis among this cohort remains small, as low as 1.4% (27). Further to the low prevalence of HHV-6 meningoencephalitis, the clinical symptoms remain nonspecific, including headache, delirium, and seizures (28), further impacting upon clinician confidence in diagnosing infection. This may have contributed to the diagnosis of two immunocompetent patients within the study cohort with HHV-6 CNS infection who received targeted anti-viral therapy with ganciclovir. Other factors which can further complicate interpretation are in CSF specimens with elevated RBC, suggestive of a traumatic lumbar puncture and possible blood contamination. Thereby, the presence may represent serum levels, rather than be representative of CNS infections (25). This may have contributed to CSF detection in 41.7% of specimens in the HHV-6 cohort in our study, which had CSF RBC levels above the ULN.

Supplementary diagnostic aids which can be utilized for HHV-6 meningoencephalitis include the presence of lymphocytic pleocytosis and elevated protein in CSF (27), although not consistently seen (25), as was similar to our study cohort with a median mononuclear cell count of 82% and elevated protein in only 25% (12/48) of specimens. Incorporation of serology into HHV-6 infection diagnostics has been infrequently reported in the literature, due to limited utility in distinguishing acute infection or reactivation from previous exposure (19). Our study had 12.5% of the HHV-6 subcohort undergo serological testing, although the diagnostic utility of one neonatal specimen is questionable unless compared to maternal serology. Furthermore, two patients with serology consistent with past infection received targeted antiviral therapy, although this may reflect the prolonged turnaround time in receiving serology results.

Serum HHV-6 quantitation can assist in differentiating active infection or reactivation; however, a high level does not consistently occur with CNS infection and can be isolated from otherwise healthy individuals (28). In contrast, digital droplet NAAT enables absolute quantitation of ciHHV-6 by fragmenting the specimen and performing multiple parallel amplification reactions concurrently (14). Although this was not performed in our study, an approximation can be made via quantitative viral load of whole blood or serum. There is a consensus in the literature that a whole blood viral load >5.5log_10_ copies/mL or serum viral load >3.5log_10_ copies/mL is suggestive of chromosomal integration (14, 24, 29, 30). However, pre-analytic factors also need to be considered prior to interpretation of viral load, including WCC count, which can significantly elevate levels (9). Of note, 50% (3/6) of patients who had serum HHV-6 quantitative NAAT performed had a viral load of >3.5log_10_ copies/mL suggestive of ciHHV-6. Overall, our study had underutilization of these supplementary tests, particularly among the subcohort of immunocompromised patients with HHV-6 detected; only two patients proceeded to have further testing, including HHV-6 quantitative NAAT. This renders interpretation challenging, given the likelihood of subclinical reactivation among immunocompromised patients and in those with severe acute illness due to another etiology (19). Hence, this emphasizes the importance of clinicians having a high pre-test probability, with concordant clinical presentation and risk factors. Pursuing supplementary testing such as quantitative HHV-6 NAAT on CSF and serum can further help clarify this, although it was not accessible in our laboratory testing network (31). This may be further prompted by the inclusion of an interpretative comment alongside HHV-6 results, suggesting detection may represent primary infection, latency, or secondary reactivation and for consideration of quantitative NAAT testing if clinically indicated.

Antiviral therapy such as ganciclovir, which targets HHV-6 DNA polymerase to inhibit viral DNA synthesis, is utilized in HHV-6 management; however, it can be associated with adverse effects such as renal impairment and bone marrow suppression (24). Other considerations including the significant financial burden of antiviral therapy and potential increased length of stay for administration of intravenous therapy. The efficacy is also poorly understood in immunocompetent pediatric populations (31), which can likely be extrapolated to immunocompetent adult patients. These concerns are similarly raised in our study, where among the 14.6% (7/48) of patients who received anti-viral therapy, three were immunocompetent hosts, including one case in a pediatric patient.

Strength of the study was the inclusion of a heterogeneous patient population, suggesting generalizability of study findings. This included analysis of CSF specimens from both pediatric and adult patients, immunocompromised patients, and inclusion of multiple regional and rural institutions. Limitations of this study include the retrospective nature and reduced sample size in some subcohorts; therefore, no power calculations were performed. Furthermore, full clinical and laboratory data were not available on all patients, in addition to lack of data regarding the prevalence of ciHHV-6 and supportive evidence such as radiological imaging to support diagnoses of HHV-6 encephalitis.

Subsequent review of these study findings provided confidence in implementing a diagnostic stewardship algorithm within our laboratory service. This involved initiating a medical microbiologist review if a non-neonate specimen had CSF WCC within normal range to determine if CSF NAAT should be performed. This included review of medical records and discussion with requesting clinician to determine the indication and underlying risk factors, e.g., immunocompromised host. Future studies worth exploring include a prospective trial following implementation of restrictive criteria for performing CSF NAAT testing on the basis of WCC and the implications on frequency of HHV-6 detection, alongside other pathogens, as well as laboratory factors, including turnaround time and cost. Development of an algorithm or tool to aid clinicians with interpretation and further investigation of HHV-6 detection on CSF specimens specific to local epidemiology, laboratory workflow practices, and available resources may also be beneficial.

### Conclusions

This study highlights the caution required when utilizing high-sensitivity, rapid diagnostic assays for the detection of CSF pathogens without laboratory stewardship. Syndromic testing is not without its challenges, and this study has highlighted that HHV-6 detection in CSF, in particular, can be prone to misinterpretation. Utilization of CSF WCC as a trigger for further review of non-neonate samples may be a useful criterion to minimize this.

## Data Availability

All data associated with this work is reported in the article.

## References

[1] Waldrop G, Zucker J, Boubour A, Radmard S, Green DA, Thakur KT. 2022. Clinical significance of positive results of the BioFire cerebrospinal Fluid FilmArray meningitis/encephalitis panel at a tertiary medical center in the United States. Arch Pathol Lab Med 146:194–200. doi:10.5858/arpa.2020-0380-OA 34086848

[2] Wang H, Zhao S, Wang S, Zheng Y, Wang S, Chen H, Pang J, Ma J, Yang X, Chen Y. 2022. Global magnitude of encephalitis burden and its evolving pattern over the past 30 years. Journal of Infection 84:777–787. doi:10.1016/j.jinf.2022.04.026 35452715

[3] Zunt JR, Kassebaum NJ, Blake N, Glennie L, Wright C, Nichols E, Abd-Allah F, Abdela J, Abdelalim A, Adamu AA, et al.. 2018. Global, regional, and national burden of meningitis, 1990–2016: a systematic analysis for the Global Burden of Disease Study 2016. Lancet Neurol 17:1061–1082. doi:10.1016/S1474-4422(18)30387-9 30507391 PMC6234314

[4] Britton PN, Dale RC, Blyth CC, Clark JE, Crawford N, Marshall H, Elliott EJ, Macartney K, Booy R, Jones CA. 2020. Causes and clinical features of childhood encephalitis: a multicenter, prospective cohort study. Clin Infect Dis 70:2517–2526. doi:10.1093/cid/ciz685 31549170

[5] Gora H, Smith S, Wilson I, Preston-Thomas A, Ramsamy N, Hanson J. 2022. The epidemiology and outcomes of central nervous system infections in Far North Queensland, tropical Australia; 2000-2019. PLoS One 17:e0265410. doi:10.1371/journal.pone.0265410 35312713 PMC8936475

[6] Greninger AL, Naccache SN, Pannaraj P, Jerome KR, Dien Bard J, Ruderman JW. 2019. The brief case: inherited chromosomally integrated human herpesvirus 6 (HHV-6) in the age of multiplex HHV-6 testing. J Clin Microbiol 57:e02016-18. doi:10.1128/JCM.02016-18 31551347 PMC6760953

[7] Aimola G, Beythien G, Aswad A, Kaufer B. 2020. Current understanding of human herpesvirus 6 (HHV-6) chromosomal integration. Antiviral Res doi:10.1016/j.antiviral.2020.104720 32044155

[8] Leong HN, Tuke PW, Tedder RS, Khanom AB, Eglin RP, Atkinson CE, Ward KN, Griffiths PD, Clark DA. 2007. The prevalence of chromosomally integrated human herpesvirus 6 genomes in the blood of UK blood donors. J Med Virol 79:45–51. doi:10.1002/jmv.20760 17133548

[9] Green DA, Pereira M, Miko B, Radmard S, Whittier S, Thakur K. 2018. Clinical significance of human herpesvirus 6 positivity on the FilmArray meningitis/encephalitis panel. Clin Infect Dis 67:1125–1128. doi:10.1093/cid/ciy288 29635334 PMC7108106

[10] Phan TL, Lautenschlager I, Razonable RR, Munoz FM. 2018. HHV-6 in liver transplantation: a literature review. Liver Int 38:210–223. doi:10.1111/liv.13506 28650593

[11] Pritchett JC, Naesens L, Montoya J, Flamand L, Lautenschlager I, Krueger GR, et al.. 2014. Treating HHV-6 Infections: The laboratory efficacy and clinical use of anti-HHV-6 agents, p 311–331. In Human Herpesviruses HHV-6A, HHV-6-B, and HHV-7. Diagnosis and Clinical management. Elsevier.

[12] The Royal Children’s Hospital Melbourne. 2019. CSF Interpretation. Available from: https://www.rch.org.au/clinicalguide/guideline_index/csf

[13] Royal College of Pathologists of Australia. 2024. Cerebrospinal Fluid Examination. Available from: https://www.rcpa.edu.au/Manuals/RCPA-Manual/Pathology-Tests/C/Cerebrospinal-fluid-examination

[14] Kestenbaum LA, Ebberson J, Zorc JJ, Hodinka RL, Shah SS. 2010. Defining cerebrospinal fluid white blood cell count reference values in neonates and young infants. Pediatrics 125:257–264. doi:10.1542/peds.2009-1181 20064869 PMC3033868

[15] Ngo Nsoga MT, Pérez-Rodriguez FJ, Mamin A, L’Huillier AG, Cherkaoui A, Kaiser L, Schibler M. 2023. Rational use of microbiological tests in the diagnosis of central nervous system infections using restrictive criteria: a retrospective study. Microbiol Spectr 11:e0317922. doi:10.1128/spectrum.03179-22 36971564 PMC10100671

[16] Leber AL, Everhart K, Balada-Llasat J-M, Cullison J, Daly J, Holt S, Lephart P, Salimnia H, Schreckenberger PC, DesJarlais S, Reed SL, Chapin KC, LeBlanc L, Johnson JK, Soliven NL, Carroll KC, Miller J-A, Dien Bard J, Mestas J, Bankowski M, Enomoto T, Hemmert AC, Bourzac KM. 2016. Multicenter evaluation of BioFire FilmArray meningitis/encephalitis panel for detection of bacteria, viruses, and yeast in cerebrospinal fluid specimens. J Clin Microbiol 54:2251–2261. doi:10.1128/JCM.00730-16 27335149 PMC5005480

[17] Myint T, Soria J, Gao Y, Conejo Castillo MR, Arora V, Ribes JA. 2025. Comparison of positive BioFire FilmArray meningitis/encephalitis (ME) panels, CSF cultures, CSF parameters, clinical presentation and in-patient mortality among patients with bacterial and fungal meningitis. Microbiol Spectr 13:e0001424. doi:10.1128/spectrum.00014-24 39714177 PMC11792450

[18] O’Halloran JA, Franklin A, Lainhart W, Burnham CA, Powderly W, Dubberke E. 2017. Pitfalls associated with the use of molecular diagnostic panels in the diagnosis of cryptococcal meningitis. Open Forum Infect Dis 4:fx242. doi:10.1093/ofid/ofx242 PMC5726458 29255738

[19] Tansarli GS, Chapin KC. 2020. Diagnostic test accuracy of the BioFire FilmArray meningitis/encephalitis panel: a systematic review and meta-analysis. Clin Microbiol Infect 26:281–290. doi:10.1016/j.cmi.2019.11.016 31760115

[20] Precit MR, Yee R, Pandey U, Fahit M, Pool C, Naccache SN, Dien Bard J. 2020. Cerebrospinal fluid findings are poor predictors of appropriate FilmArray meningitis/encephalitis panel utilization in pediatric patients. J Clin Microbiol 58:e01592-19. doi:10.1128/JCM.01592-19 31852767 PMC7041564

[21] Patel R, Fang FC. 2018. Diagnostic stewardship: opportunity for a laboratory-infectious diseases partnership. Clin Infect Dis 67:799–801. doi:10.1093/cid/ciy077 29547995 PMC6093996

[22] Cunha BA. 2006. Distinguishing bacterial from viral meningitis: the critical importance of the CSF lactic acid levels. Intensive Care Med 32:1272–1273; doi:10.1007/s00134-006-0210-x 16770614

[23] Chakrabarti P, Warren C, Vincent L, Kumar Y. 2018. Outcome of routine cerebrospinal fluid screening for enterovirus and human parechovirus infection among infants with sepsis-like illness or meningitis in Cornwall, UK. Eur J Pediatr 177:1523–1529. doi:10.1007/s00431-018-3209-8 30022279

[24] Pandey U, Greninger AL, Levin GR, Jerome KR, Anand VC, Dien Bard J. 2020. Pathogen or bystander: clinical significance of detecting human herpesvirus 6 in pediatric cerebrospinal fluid. J Clin Microbiol 58:e00313-20. doi:10.1128/JCM.00313-20 32102858 PMC7180253

[25] Chia XT, Wong HLM, Loh JS. 2024. Human herpesvirus-6 infection in a critically ill and immunocompetent patient: a case report. J Med Case Rep 18. doi:10.1186/s13256-024-04387-5 PMC10905906 38424575

[26] Raouf MME, Ouf NM, Elsorady MAS, Ghoneim FM. 2023. Human herpesvirus-6 in hematopoietic stem cell transplant recipients: a prospective cohort study in Egypt. Virol J 20:20. doi:10.1186/s12985-023-01980-w 36739398 PMC9899109

[27] Li N, Zhang R, Wang J, Zhu X, Meng F, Cao Y, Wang G, Yang Y. 2024. Case report: Acute HHV6B encephalitis/myelitis post CAR-T cell therapy in patients with relapsed/refractory aggressive B-cell lymphoma. Front Neurol 15:1334000. doi:10.3389/fneur.2024.1334000 38487325 PMC10937551

[28] Rebechi MT, Bork JT, Riedel DJ. 2021. HHV-6 encephalitis after chimeric antigen receptor T-cell therapy (CAR-T): 2 case reports and a brief review of the literature. Open Forum Infect Dis 8:ofab470. doi:10.1093/ofid/ofab470 34738024 PMC8562470

[29] Pellett PE, Ablashi DV, Ambros PF, Agut H, Caserta MT, Descamps V, Flamand L, Gautheret-Dejean A, Hall CB, Kamble RT, et al.. 2012. Chromosomally integrated human herpesvirus 6: questions and answers. Rev Med Virol 22:144–155. doi:10.1002/rmv.715 22052666 PMC3498727

[30] Ward KN, Leong HN, Thiruchelvam AD, Atkinson CE, Clark DA. 2007. Human herpesvirus 6 DNA levels in cerebrospinal fluid due to primary infection differ from those due to chromosomal viral integration and have implications for diagnosis of encephalitis. J Clin Microbiol 45:1298–1304. doi:10.1128/JCM.02115-06 17229866 PMC1865851

[31] Crawford JR, Kadom N, Santi MR, Mariani B, Lavenstein BL. 2007. Human herpesvirus 6 rhombencephalitis in immunocompetent children. J Child Neurol 22:1260–1268. doi:10.1177/0883073807307086 18006954

